# Transportation/Fuels: Souped-Up Yeast

**DOI:** 10.1289/ehp.113-a231a

**Published:** 2005-04

**Authors:** Carol Potera

The high cost of oil makes ethanol and other alternative fuels increasingly attractive. Proponents of ethanol point to corn, wheat, and other food crops as renewable feedstocks for producing the fuel. However, critics contend that diverting food crops for ethanol production is economically unsound, and that the irrigation, pesticides, and diesel fuel used to produce these crops poses an environmental burden. A new solution converts agricultural waste such as cornstalks and wheat straw into ethanol. Molecular biologist Nancy Ho of Purdue University’s Laboratory of Renewable Resources Engineering spent 20 years perfecting the method, which has been nonexclusively licensed to Canadian enzyme manufacturer Iogen to make ethanol in an environmentally friendly plant.

Ethanol is produced through fermentation of the glucose found in plant matter. The yeast *Saccharomyces*—used for centuries to make wine, beer, and bread—is the most efficient microorganism for fermenting glucose to ethanol. Food crops such as corn and wheat are especially suitable for ethanol production because the glucose in their kernels is readily fermentable by *Saccharomyces*. In contrast, the cellulose found in cornstalks and other types of cellulosic biomass contains not only glucose, but also the sugar xylose, which *Saccharomyces* cannot convert to ethanol because it lacks the enzymes to do so.

Glucose and xylose can be fermented separately, but it’s a costly process. Some manufacturers do convert just the glucose in waste feedstocks to ethanol, but production is very low. If the xylose fermentation hurdle could be overcome, the waste material left in the cornfield after harvest could produce 4–5 billion gallons of ethanol annually, says Ho.

Ho’s solution was to create a genetically modified strain of *Saccharomyces* that simultaneously ferments both glucose and xylose to ethanol. Some bacteria contain the enzyme xylose isomerase, which ferments xylose to ethanol in one step. In the 1980s, Ho first cloned and inserted a bacterial gene for xylose isomerase into *Saccharomyces*, only to discover that the enzyme did not function inside the yeast. Her second approach proved successful, but required several enzymes and complex steps—in short, Ho cloned three genes from another yeast and inserted them into *Saccharomyces*. They act in a pathway to convert xylose into xylitol, xylulose, and xylulose-5-phosphate, before eventually producing ethanol. Ho also tinkered with the enzymes to make them operate faster. This work is described in the spring 2004 issue of *Applied Biochemistry and Biotechnology*.

The novel method raises the yield of ethanol by 40%, compared to fermenting only the glucose in cornstalks and related materials. One of the resulting recombinant yeasts, named 424A(LNH-ST), is now being used by the Ottawa-based Iogen to produce ethanol using wheat straw obtained from nearby farms. The fuel is sold under the trade name EcoEthanol™.

Iogen had tried recombinant yeasts and bacteria designed by other scientists, but they performed poorly when scaled up for industrial production. “The Purdue yeast is the best we’ve tested,” says chemical engineer Jeff Tolan, Iogen’s manager of process research and development. “The Purdue yeast is as easy to work with as making bread at home.” Although the Iogen plant has used only wheat straw as a feedstock, the Purdue yeast could efficiently convert xylose from cornstalks, wood chips, and cardboard to ethanol in their processing facility.

The Iogen plant makes about 75 gallons of ethanol per ton of straw. About two-thirds of the straw is fermented to ethanol. The remainder, which is primarily lignin, is burned for fuel at a local pulp mill. For a full-scale ethanol plant, the lignin could be used to generate power for the plant. “A full-scale plant could be run without any net burning of fossil fuel,” says Tolan. In addition, EcoEthanol reduces the net generation of greenhouse gases, because the plants being grown for feedstock recycle the carbon dioxide released into the atmosphere when the fuel is burned. Iogen plans to build a full-scale commercial facility that will produce 50 million gallons of EcoEthanol yearly.

## Figures and Tables

**Figure:**
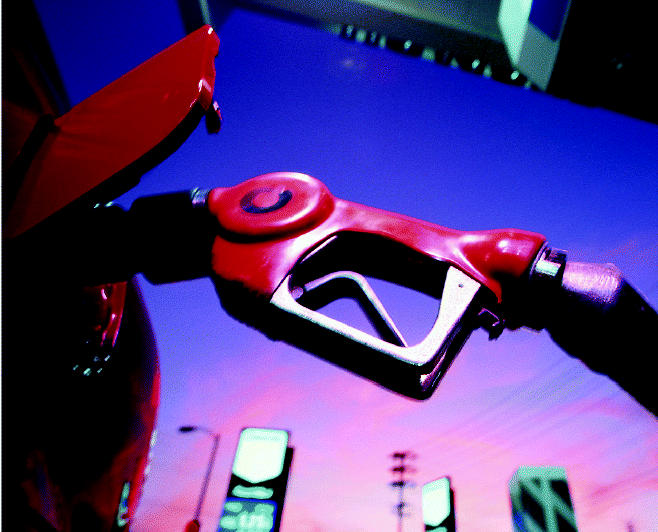
**Pump up the volume.** A novel method increases the amount of ethanol that can be derived from agricultural waste.

